# Use of Cottonseed Meal in Feeding Yellow-Feathered Broilers: Effects on Performance Parameters, Digestibility and Meat Quality

**DOI:** 10.3390/vetsci12050416

**Published:** 2025-04-27

**Authors:** Xiaohang Nie, Xiahan Wei, Weidong Niu, Fengming Li, Jiang Yuan, Gang Lv, Yong Chen, Jiancheng Liu

**Affiliations:** 1 Research Center for Biological Feed and Animal Gut Health, College of Animal Science, Xinjiang Agricultural University, Urumqi 830052, China; 17630848120@163.com (X.N.); hanhan13462017360@163.com (X.W.); nwd163com@163.com (W.N.); lifming@163.com (F.L.); xjaucy@163.com (Y.C.); 2 Xinjiang Tycoon Group Co., Ltd., Changji 831199, China; 18199843513@163.com (J.Y.); j17799683498@163.com (G.L.)

**Keywords:** apparent metabolizability, broiler growth, serum biochemical indexes, slaughter performance

## Abstract

This study investigated the effects of replacing soybean meal (SBM) with high-protein cottonseed meal (CSM) and de-phenolized cottonseed meal (DPCSM) on growth performance, apparent nutrient digestibility, serum biochemical parameters, slaughter traits, and meat quality in yellow-feathered broilers. A total of 5760 one-day-old male Liangfeng Hua broilers were randomly divided into five groups with eight replicates per group. The control group was fed a corn-SBM diet (SBM group), while the CSM_50_, CSM_100_, DPCSM_50_, and DPCSM_100_ groups replaced 50% and 100% of the equivalent protein from SBM with CSM and DPCSM, respectively. Compared to the control group, the CSM_50_ and DPCSM_50_ groups showed no significant negative effect on growth performance; however, dietary calcium digestibility was significantly reduced on day 21. Furthermore, CSM_100_ and DPCSM_100_ adversely impacted growth performance, significantly reducing crude fat digestibility and increasing serum urea nitrogen levels on day 42. The broilers in these groups also had reduced pre- and post-slaughter body weights, while those in the CSM_50_ and DPCSM_100_ groups exhibited decreased half-clearance rates. In addition, the CSM_100_ group had significantly increased pectoral muscle drip loss and a* (24 h) values. pH values measured at 45 min and 24 h were significantly elevated in the DPCSM_50_ and DPCSM_100_ groups. In conclusion, the replacement of SBM with CSM and DPCSM in yellow-feathered broiler diets should be limited to 50%.

## Simple Summary

Soybean meal is widely used as high-quality protein in poultry feed, but its high cost affects the sustainability of the farming industry. Scientists have attempted to replace soybean meal with more economical cotton meal, but traditional cotton meal contains toxins (e.g., gossypol) that negatively impact animal health. In this study, processing techniques were improved to produce high-protein cotton meal and de-phenolized cotton meal. The new feed was tested in 5760 yellow-feathered broilers at different substitution ratios (50% and 100%). The results revealed that when 50% soybean meal was replaced with modified cotton meal, the broiler growth rate and meat quality were not affected and toxin residues were not exceeded; however, complete substitution reduced feed digestibility, led to slower body weight gain, abnormal blood health indicators, and increased muscle water loss. These findings indicated that up to 50% of the soybean meal can be replaced by the modified cotton meal in yellow-feathered broilers without compromising their health parameters. These results provide farmers with an environmentally friendly feed option, reduce dependence on imported soybean meal, and promote the efficient use of agricultural resources and sustainable development.

## 1. Introduction

Soybean meal (SBM) is valued for its high nutritional quality and balanced amino acid profile and is a widely utilized plant-derived protein source in poultry feed [1]. However, the escalating cost of SBM poses a significant challenge to the sustainable development of the livestock industry [2]. Consequently, sustainable alternatives are required to fulfill the demand for plant-based protein in animal nutrition [3].

Globally, cottonseed meal (CSM) is the second-largest plant-based protein source for animal feed after soybean meal (SBM) [4], with an annual production of 10–11 million tons worldwide [5]. Recognized for its high protein content, relatively balanced amino acid profile, and wide availability, CSM is a promising plant protein source [6]. Nonetheless, its utilization in poultry diets is limited by harmful nutritional factors such as gossypol, cyclopropenoid fatty acids, phytic acid, and non-starch polysaccharides [7]. Notably, the free gossypol (FG) content limits its application in poultry production [7]. Free gossypol (FG) has been demonstrated to adversely affect animal growth performance, metabolism, and organ function [8,9,10].

Still, physical [11], chemical [12,13,14], and biological [15,16,17] methods have proven effective in reducing FG content, thereby enhancing the nutritional and functional value of cottonseed meal (CSM). Reviews by Nagalakshmi et al. [7] and Świątkiewicz et al. [18] reported that up to 15% of CSM in poultry diets—with FG levels below 100 mg/kg—can be safely supplemented to soybean meal (SBM). Moreover, Yu et al. [19] proposed that low-gossypol cottonseed meal (CSM) could serve as a complete SBM replacement in monogastric diets. Subsequent studies by Abdallh et al. [20,21] further demonstrated that dietary inclusion of 18% CSM in Ross 308 male broilers (1–35 days post-hatch) effectively replaced 90% of SBM without compromising growth performance or feed efficiency.

Advances in modern cottonseed processing technologies have significantly upgraded CSM’s nutritional profile. Current methodologies utilize methanol/ethanol-based secondary solvent extraction, yielding detoxified cottonseed meal (DPCSM) with reduced free gossypol (FG) content (<400 mg/kg) and elevated crude protein levels (60–80%) [22,23,24]. These modifications enhance both the nutritional density and safe inclusion thresholds of DPCSM, positioning it as a cost-effective, functionally equivalent substitute for SBM in feed formulations. Despite these advancements, the practical application can be further optimized. A limited number of peer-reviewed articles have been published on the efficacy of high-protein CSM variants and DPCSM in broiler production systems, particularly concerning long-term metabolic impacts and nutrient bioavailability. Hence, further zootechnical evaluations are warranted to validate the complete replacement potential of these novel protein sources across diverse poultry genotypes and production phases.

This study aimed to investigate the effects of high-ratio (50% and 100%) replacement of soybean meal (SBM) with high-protein cottonseed meal (CSM) and de-phenolized CSM (DPCSM) on growth performance, dietary apparent metabolizability, blood biochemical parameters, slaughter traits, and meat quality in yellow-feathered broilers.

## 2. Materials and Methods

### 2.1. Ethical Statement

All animal experiments were conducted according to the relevant national guidelines and were approved by the Animal Welfare Care and Use Committee of Xinjiang Agricultural University (Xinjiang, China) (Animal Protocol Number: 2024005).

### 2.2. Test Material

The high-protein cottonseed meal (CSM) and de-phenolized CSM (DPCSM) used in this trial were sourced from the Xinjiang Taikun Group. The analyzed crude protein content of CSM was 60.32%, with a free gossypol (FG) level of 675.7 mg/kg. DPCSM was produced from CSM through secondary methanol extraction of gossypol followed by low-temperature desolventization, yielding a product containing 60.86% crude protein and 387.6 mg/kg FG.

### 2.3. Experimental Design and Diets

A total of 5760 one-day-old male Liangfeng Hua yellow-feathered broilers (average body weight: 39.58 ± 0.26 g) were randomly assigned to 5 groups with 8 replicates per group and 144 birds per replicate. The control group received a corn-soybean meal (SBM) basal diet, while the experimental groups (CSM_50_, CSM_100_, DPCSM_50_, DPCSM_100_) were fed diets in which 50% or 100% of SBM in the control diet was replaced isoproteically with high-protein CSM or de-phenolized CSM (DPCSM). The trial was conducted in three phases: days 1–21, 22–42, and 43–63. Diets were formulated according to the Nutritional Requirements of Yellow Chicken (NY/T 3645-2020). The detailed compositions and nutrient levels are listed in Table 1. The birds were housed in a three-tier cage system with partitioned pens and were allowed free access to feed and water. Lighting, vaccination, and routine management were set according to the guidelines of the Feeding Management Regulations of Yellow-Feathered Chicken (NY/T 1871-2010).

### 2.4. Measurements

On days 22, 43, and 64, fasted body weight (WG) and feed intake were recorded for each group in the morning. Growth performance metrics, including average daily gain (ADG), average daily feed intake (ADFI), and feed-to-gain ratio (F/G), were calculated for each phase (1–21, 22–42, and 43–63 days) on a per-replicate basis.

Dietary apparent metabolizability was determined using the total fecal collection method [25]. Daily feed intake and residues were weighed four days prior to the end of each phase. Subsequently, feces were collected, homogenized, and subsampled (2–10% of total weight). To minimize ammonia nitrogen loss, 10 mL of 10% hydrochloric acid was added per 100 g of fresh feces. The dry matter (DM), crude protein (CP), crude fat (EE), ash, calcium (Ca), and phosphorus (P) in feed and feces were analyzed following Association of Official Analytical Chemists (AOAC) [26]. The Apparent metabolizability values of the diets were determined according to the equations proposed by Matterson et al. [27] (1):

(1) $$Apparent\;metabolizability\left(\%\right)=\frac{\left(Nutrient\;intake-Fecal\;nutrient\;excretion\right)}{Nutrient\;intake}\times 100$$

On day 64, blood was collected from one randomly selected bird per replicate via wing vein puncture. Serum levels of alanine aminotransferase (ALT), aspartate aminotransferase (AST), total protein (TP), albumin (ALB), globulin (GLB), urea nitrogen (BUN), triglycerides (TG), total cholesterol (T-CHO), and glucose (GLU) were measured using an automated biochemical analyzer (TBA-FX8, Medical Imaging System, Canon Medical Systems Corporation, Ōta, Tokyo, Japan).

Birds closest to the group average weight were slaughtered on day 64. Slaughter performance (dressing percentage, semi-eviscerated carcass yield, eviscerated carcass yield, breast muscle yield, thigh muscle yield, and abdominal fat percentage) was evaluated according to the Performance Terminology and Measurement Methods for Poultry (NY/T 823-2020).

Meat quality assessment was conducted according to the method described by de Moraes Pinto, L.A. et al. [28]. Samples were obtained from the ipsilateral pectoralis major muscle for analysis. Color attributes were measured using a portable chroma meter (CR-10, Chroma Meter, Konica Minolta Inc., Chiyoda, Tokyo, Japan) to determine lightness (L*), redness (a*), and yellowness (b*). pH values were recorded at 45 min and 24 h postmortem with a portable pH meter (Model H1981036, pH Meter, HANNA Instruments, Inc., Woonsocket, RI, USA). Shear force, an indicator of tenderness, was quantified using a shear force tester (C-LM3B, Biomechanical Testing System, College of Engineering, Northeast Agricultural University, Harbin, Heilongjiang Province, China). For cooking loss determination, meat samples (2.0 cm × 3.5 cm × 5.0 cm) were weighed (*W*1) within 1 h postmortem, heated in an 80 °C water bath for 10 min, blotted dry, and reweighed (*W*2). Cooking loss was calculated as follows:

$$Cooking\;loss\;(\%)=\frac{W1-W2}{W1}\times 100$$

For drip loss analysis, samples of identical dimensions were weighed (*W*3), stored at 4 °C for 24 h, and reweighed (*W*4). Drip loss was determined using the following:

$$Drip\;loss\;(\%)=\frac{W3-W4}{W3}\times 100$$

### 2.5. Statistical Analyses

Data were expressed as the mean ± standard deviation (SD) and analyzed using the SPSS software program (version 27.0; IBM Corp., Armonk, NY, USA). One-way analysis of variance (ANOVA) was used to test for differences among treatments, and Tukey’s test was applied for multiple comparisons. Statistical significance was set at *p* < 0.05.

## 3. Results

### 3.1. Growth Performance

During the starter phase (1–21 days), no significant differences in WG and ADG were observed between the CSM_100_ and DPCSM_100_ groups (*p* > 0.05), as shown in Table 2; however, both were significantly lower than the SBM group (*p* < 0.05). In addition, no differences in WG or ADG were observed between CSM_100_ and CSM_50_ (*p* > 0.05), whereas DPCSM_100_ exhibited significantly lower WG and ADG compared to DPCSM_50_ (*p* < 0.05). The CSM_50_, DPCSM_50_, and SBM groups showed comparable WG and ADG values (*p* > 0.05). Nonetheless, ADFI and F/G demonstrated no significant difference across groups (*p* > 0.05). In the grower phase (22–42 days), the CSM_100_ groups displayed significantly lower WG, ADFI, and ADG values compared to SBM and CSM_50_ (*p* < 0.05); still, no differences were observed compared to the DPCSM_100_ group (*p* > 0.05). The DPCSM_100_ group showed reduced WG and ADFI values (*p* < 0.05) compared to SBM and DPCSM_50_, though ADG remained unaffected (*p* > 0.05). Moreover, the CSM_50_, DPCSM_50_, and SBM groups exhibited similar WG, ADFI, and ADG values (*p* > 0.05). Nevertheless, F/G showed no significant difference among groups (*p* > 0.05). During the finisher phase (43–63 days), CSM_100_ and DPCSM_100_ had comparable WG values (*p* > 0.05) but were significantly lower than SBM (*p* < 0.05). No differences in WG were observed between CSM_100_ and CSM_50_ or between DPCSM_100_ and DPCSM_50_ (*p* > 0.05). Additionally, the ADFI, ADG, and F/G values were consistent across all groups (*p* > 0.05). Over the overall trial period (1–63 days), ADFI and ADG in the CSM_100_ and DPCSM_100_ groups were significantly lower than SBM (*p* < 0.05) but comparable to each other (*p* > 0.05). The CSM_100_ group exhibited reduced ADFI compared to the CSM_50_ group (*p* < 0.05), whereas the DPCSM_100_ group showed no differences in ADFI or ADG compared to the DPCSM_50_ group (*p* > 0.05). Furthermore, the CSM_50_, DPCSM_50_, and SBM groups maintained similar ADFI and ADG (*p* > 0.05). F/G did not differ among treatments (*p* > 0.05).

### 3.2. Dietary Apparent Metabolizability

As shown in Table 3, at 21 days of age, the calcium (Ca) apparent digestibility was significantly lower in the CSM_50_ group compared to the SBM and CSM_100_ groups (*p* < 0.05) but did not differ from the DPCSM_50_ group (*p* > 0.05). Similarly, the DPCSM_50_ group exhibited reduced Ca digestibility compared to the SBM group (*p* < 0.05), also showing no significant difference compared to the DPCSM_100_ group (*p* > 0.05). Notably, no significant variations in Ca digestibility were detected among the CSM_100_, DPCSM_100_, and SBM groups (*p* > 0.05). Dry matter (DM), organic matter (OM), gross energy (GE), crude protein (CP), crude fat (EE), and phosphorus (P) digestibility showed no significant differences across groups (*p* > 0.05). At 42 days, a significant decrease in GE digestibility was observed in the DPCSM_100_ group compared to other experimental groups (*p* < 0.05). EE digestibility decreased in the DPCSM_100_ group compared to the SBM and CSM_50_ groups (*p* < 0.05) but remained comparable to the CSM_100_ and DPCSM_50_ groups (*p* > 0.05). The CSM_100_ and DPCSM_50_ groups also showed reduced EE digestibility compared to the SBM group (*p* < 0.05) but not the CSM_50_ group (*p* > 0.05). In addition, the CSM_100_ group showed a lower P digestibility than the SBM and DPCSM_100_ groups (*p* < 0.05) but was similar to the CSM_50_ group (*p* > 0.05). Conversely, the DPCSM_100_, DPCSM_50_, and SBM groups exhibited comparable P digestibility (*p* > 0.05). DM, OM, CP, and Ca digestibility remained consistent across all groups (*p* > 0.05). By 63 days, no significant differences were observed in DM, OM, GE, CP, EE, Ca, or P digestibility among the various groups (*p* > 0.05).

### 3.3. Blood Biochemical Parameters

As shown in Table 4, no significant differences (*p* > 0.05) were observed in the serum levels of total protein (TP), albumin (ALB), globulin (GLB), urea nitrogen (BUN), triglycerides (TG), total cholesterol (T-CHO), alanine aminotransferase (ALT), aspartate aminotransferase (AST), or glucose (GLU) among the experimental groups at 21 days of age. At 42 days of age, the DPCSM_50_ group exhibited significantly higher serum TG levels compared to the SBM and DPCSM_100_ groups (*p* < 0.05), though no significant difference was found between the DPCSM_50_ and CSM_50_ groups (*p* > 0.05). Markedly, lower serum BUN levels were observed in the CSM_50_ group compared to the CSM_100_ group (*p* < 0.05), but no differences were observed between the CSM_50_ group and the DPCSM_50_ or SBM groups (*p* > 0.05). The DPCSM_50_ group revealed significantly reduced BUN levels relative to the DPCSM_100_ group (*p* < 0.05), whereas the BUN levels in the DPCSM_50_ group remained comparable to those in the SBM group (*p* > 0.05). Both the CSM_100_ and DPCSM_100_ groups displayed significantly elevated BUN levels compared to the SBM group (*p* < 0.05), though no difference was detected between these two groups (*p* > 0.05). No significant variations (*p* > 0.05) were observed in TP, ALB, GLB, T-CHO, ALT, AST, and GLU levels across all groups. By 63 days of age, the serum TG levels in the DPCSM_100_ group were significantly higher than those in the SBM and CSM_100_ groups (*p* < 0.05), but no difference was observed between the DPCSM_100_ and DPCSM_50_ groups (*p* > 0.05). All experimental groups exhibited significantly higher serum GLU levels compared to the SBM group (*p* < 0.05), with no intergroup differences (*p* > 0.05). TP, ALB, GLB, BUN, T-CHO, ALT, and AST levels remained consistent across all treatments (*p* > 0.05).

### 3.4. Slaughter Performance

As shown in Table 5, the semi-eviscerated yield of the CSM_50_ group was significantly lower than that of the SBM group (*p* < 0.05) but similar to the CSM_100_ and DPCSM_50_ groups (*p* > 0.05). The DPCSM_100_ group displayed a reduced semi-eviscerated yield compared to the SBM group (*p* < 0.05), though no differences were detected compared to the DPCSM_50_ or CSM_100_ groups (*p* > 0.05). The slaughter rate, fully eviscerated yield, breast muscle yield, leg muscle yield, and abdominal fat percentage showed no significant difference among groups (*p* > 0.05).

### 3.5. Meat Quality

As shown in Table 6, the drip loss rate in the CSM_100_ group was significantly higher than in the SBM and DPCSM_100_ groups (*p* < 0.05) but comparable to the CSM_50_ group (*p* > 0.05). The pH at 45 min demonstrated no significant difference between the DPCSM_50_ and DPCSM_100_ groups (*p* > 0.05); however, both were significantly higher than the CSM_50_, CSM_100_, and SBM groups (*p* < 0.05). At 24 h, the pH in the DPCSM_100_ group was significantly higher than those in the SBM, CSM_100_ and DPCSM_50_ groups (*p* < 0.05). Moreover, the DPCSM_50_ group exhibited a higher pH at 24 h compared to the SBM group and the CSM_50_ group (*p* < 0.05). The a* value at 45 min in the DPCSM_50_ group was significantly higher than in the CSM_50_ group (*p* < 0.05) but similar to the DPCSM_100_ and SBM groups (*p* > 0.05). At 24 h, the a* value in the CSM_100_ group was higher than in the CSM_50_ and SBM groups (*p* < 0.05) but showed no significant difference from the DPCSM_100_ group (*p* > 0.05). Cooking loss, shear force, L* (45 min and 24 h), and b* (45 min and 24 h) showed no significant differences among groups (*p* > 0.05).

## 4. Discussion

Cottonseed meal (CSM) shows promising developmental potential as a plant-based protein feed resource and has been widely used to replace soybean meal (SBM) in poultry diets to reduce feed costs [7,18]. However, excessive inclusion of CSM in the poultry diet may lead to adverse effects such as a reduced growth rate, decreased feed intake, inefficient feed conversion, and increased mortality due to elevated free gossypol (FG) levels. It is noteworthy that other antinutritional factors in CSM, such as tannins, may directly affect feed palatability due to their sensory properties (e.g., astringency and bitterness), potentially causing chickens to avoid consuming the diet because of undesirable taste or olfactory cues [29]. The tolerance of poultry to CSM is influenced by multiple factors, including FG content, source, inclusion level, protein quality, breed, age, and dietary lysine and iron concentrations [10]. This study revealed that replacing 50% of SBM with CSM or de-phenolized CSM (DPCSM) in yellow-feathered broiler diets showed no significant impact on growth performance. However, full replacement (100%) significantly reduced body weight gain (WG), average daily gain (ADG), and average daily feed intake (ADFI). These results indicate that the complete substitution of SBM with CSM/DPCSM compromises production performance, which is consistent with the findings of Adeyemo et al. [30]. This decreased production performance may be attributed to antinutritional factors such as gossypol in CSM/DPCSM, which impair nutrient digestion and absorption. Free gossypol binds to proteolytic enzymes in the gastrointestinal tract, inhibiting pepsin and trypsin activity, reducing protein digestibility, and potentially damaging digestive tissues, thereby impairing growth [31]. Additionally, FG inhibits gastrin release, suppressing appetite and further reducing feed intake [7]. This appetite suppression, combined with potential sensory aversion to FG-related bitterness or other off-flavors in CSM-containing diets, could synergistically contribute to the observed reduction in feed intake [29]. Reduced feed intake is a primary symptom of SBM replacement with CSM [32,33], leading to insufficient nutrient intake and subsequent declines in ADG and WG. Our results revealed that the full replacement of SBM with CSM/DPCSM significantly reduced WG and ADG during days 1–21, and WG, ADFI, and ADG during days 22–42. Conversely, WG was significantly decreased during days 43–63, though ADFI and ADG remained unaffected. These findings suggest that the negative impacts of CSM/DPCSM are more pronounced in younger broilers, which is likely attributed to immature digestive systems and lower amino acid utilization efficiency from CSM compared to SBM [34]. Haribhau et al. [35] similarly reported that 10% CSM inclusion reduced body weight gain in 1–28-day-old broilers but had no effect during 29–42 days. FG accumulation in tissues may further contribute to reduced ADFI and ADG over the entire 1–63-day period. Notably, DPCSM exhibited stronger negative effects compared to CSM, which may be due to residual FG or the conversion of bound gossypol to FG in vivo [36]. Furthermore, methanol-based extraction during DPCSM processing may also denature proteins, impairing digestibility. Chen et al. [23] confirmed that even de-phenolized cottonseed protein concentrate (62.98% CP) replacing 50% SBM in Arbor Acres broiler diets resulted in reduced ADG and ADFI, highlighting the limitations of FG removal strategies. In conclusion, the application of CSM or DPCSM replacement in poultry diets varies based on breed, age, and inclusion levels. While partial replacement (≤50%) is feasible, full substitution (100%) significantly compromises performance, particularly in younger birds, due to FG’s antinutritional effects and potential protein quality degradation.

The apparent metabolizability of dietary nutrients serves as a critical indicator for evaluating nutrient utilization efficiency in poultry, reflecting the proportion of ingested nutrients that are effectively assimilated and retained during metabolic processes [37]. The incorporation of cottonseed meal (CSM) into broiler diets significantly impairs nutrient digestion and absorption due to its antinutritional components, including free gossypol, phytic acid, and non-starch polysaccharides (NSPs) [33]. Free gossypol inhibits pepsin and trypsin activity, thereby reducing protein digestibility [38], whereas its iron-binding capacity disrupts hemoglobin synthesis and induces iron-deficiency anemia [39]. Phytic acid chelates essential minerals (e.g., Zn, Ca, Cu, Mg, Mn, Fe) and phosphorus, forming insoluble phytate complexes that diminish mineral bioavailability [40]. Additionally, phytic acid inhibits digestive enzymes such as proteases, amylases, and lipases, further limiting nutrient absorption [41]. NSPs interfere with the breakdown of proteins, lipids, and carbohydrates, exacerbating nutrient utilization challenges. Recent studies demonstrate the metabolic consequences of CSM substitution. Ashayerizadeh et al. [42] replaced 50% soybean meal (SBM) with CSM in Ross 308 broilers, resulting in reduced ileal apparent digestibility of crude protein (66.5% vs. 74.8%) and crude fat (75.14% vs. 80.97%). Exogenous enzyme supplementation has emerged as an effective strategy to optimize CSM utilization. Tavares-Samay et al. [4] reported that 25% CSM substitution in Cobb-500 broiler diets altered metabolic energy patterns and reduced calcium/phosphorus retention, though phytase supplementation improved mineral metabolizability. Similarly, Safari et al. [43] revealed that 30% CSM replacement in Ross 308 broilers with 0.02% protease supplementation maintained production performance while enhancing protein digestibility. Furthermore, Abdallh et al. [21] demonstrated dose-dependent improvements in arginine, glutamic acid, and threonine digestibility with CSM substitution, though phosphorus digestibility declined. Notably, xylanase-β-glucanase complexes in high-CSM diets enhanced crude protein, starch, methionine, calcium, and manganese utilization while stimulating endogenous lipase activity. In the present study, compound enzymes (protease + NSPase) were added to CSM-based diets to enhance nutrient availability and enable high SBM replacement. The results revealed decreased the apparent metabolizability of calcium (21-day) and ether extract (42-day) in yellow-feathered broilers fed CSM/DPCSM diets. These findings may result from altered dietary calcium–phosphorus ratios and reduced calcium content following SBM substitution. Calcium–phosphorus homeostasis is critical for skeletal development of fast-growing broilers. Disrupted calcium levels can impair the structural integrity of bone growth plates, potentially leading to metabolic bone disorders such as rachitis and tibial dyschondroplasia [44]. These pathologies are particularly detrimental to modern broiler strains that have been selected for rapid growth rates, as their high metabolic demands exacerbate their susceptibility to skeletal abnormalities under suboptimal mineral conditions [44]. The low ether extract levels in high-protein CSM and DPCSM were attributed to deflocking, decortication, and dual solvent extraction (methanol/hexane), which likely contributed to reduced fat metabolizability. These findings highlight the necessity of enzymatic interventions to mitigate the antinutritional effects of CSM and optimize its application in poultry nutrition.

Serum biochemical parameters reflect metabolic status and physiological changes in animals, particularly in response to nutrient utilization and organ function. Previous studies have demonstrated that excessive free gossypol (FG) intake from CSM adversely affects blood biochemical profiles in poultry [7]. Specifically, Adeyemo [45] reported reduced serum total protein (TP) and globulin (GLB) levels in CSM-fed broilers. Similarly, Zeng et al. [9,46] observed linear decreases in serum TP, albumin (ALB), and GLB concentrations in ducks as dietary CSM inclusion was increased (FG: 36, 75, 111, and 153 mg/kg), accompanied by exacerbated hepatic damage. Liver injury was found at FG levels of 320 mg/kg, as evidenced by elevated serum alanine aminotransferase (ALT) and aspartate aminotransferase (AST) levels [10]. Yu et al. [47] further reported that increasing CSM levels (FG: 56–222 mg/kg) in gosling diets resulted in a linear reduction in serum TP and ALB levels and elevated uric acid concentrations. However, some studies suggest that moderate FG levels may not compromise blood biochemistry. Wang et al. [48] found no alterations in serum ALT or AST activities in Arbor Acres (AA) broilers fed diets containing 7.5–8.9% CSM (FG: 61.5–73 mg/kg) at 21 or 42 days. Yu et al. [19] found that the serum total cholesterol (TC) and triglyceride (TG) levels in 70-day-old Jiangnan geese fed with CSM (26.91% inclusion; FG: 40.37 mg/kg) as a full SBM replacement were unaffected. Moreover, Sun et al. [49] demonstrated that substituting soybean meal (SBM) with 5%, 8%, and 16% cottonseed meal (CSM; free gossypol = 58, 92.8, and 185.6 mg/kg, respectively) in Arbor Acres (AA) male broiler diets (28–42 days of age) elicited no significant alterations in serum biochemical parameters, including total protein (TP), albumin (ALB), globulin (GLB), urea nitrogen (UN), total cholesterol (TC), triglycerides (TG), high-density lipoprotein cholesterol (HDL-C), low-density lipoprotein cholesterol (LDL-C), alanine aminotransferase (ALT), and aspartate aminotransferase (AST). Collectively, these findings indicate that dietary FG concentrations ≤185.6 mg/kg do not adversely affect protein synthesis or lipid metabolism pathways in broilers. In the present study, high-protein CSM and de-phenolized CSM (DPCSM) substitution in yellow-feathered broiler diets resulted in elevated serum TG (42 and 63 days), BUN (42 days), and glucose (GLU, 63 days) levels, indicating disruptions in lipid, protein, and energy metabolism. In this study, the observed elevated BUN may reveal compromised renal function, as it is a key biomarker of nitrogen metabolism and renal excretion efficiency [50]. Prolonged elevation of BUN levels in fast-growing broilers could impose additional metabolic stress on kidneys, potentially leading to tubular dysfunction or glomerular damage over time [51]. These effects may be mediated by increased antinutritional factors (FG and cyclopropenoid fatty acids) interfering with lipid metabolism, leading to TG accumulation. Elevated BUN suggests reduced protein utilization, potentially contributing to weight loss. Furthermore, while direct renal pathology markers (e.g., serum creatinine) were not measured in our study, the observed metabolic disturbances highlight the need for future investigations to assess CSM-induced nephrotoxicity, particularly in poultry breeds with high growth rates and susceptibility to renal overload. Notably, Chen et al. [23] reported no changes in serum TG or BUN, whereas ALT and alkaline phosphatase (ALP) were increased in AA broilers fed cottonseed protein concentrate (62.98% CP) replacing 25 or 50% SBM, implying latent hepatic stress. These findings highlight that the impact of CSM/DPCSM on serum biochemistry varies based on FG levels, poultry species, and substitution ratios. While partial replacement may be tolerated, high inclusion disrupts metabolic homeostasis. Therefore, formulations should be balanced to mitigate antinutritional effects.

Carcass traits, particularly breast and thigh muscle yields, are critical economic indicators in broiler production. Optimizing these parameters in yellow-feathered broilers remains a key research focus, with significant implications for enhancing industry profitability and meeting market demands. Previous studies revealed that free gossypol (FG) in cottonseed meal (CSM) may impair protein metabolism by binding to blood proteins, potentially affecting muscle deposition and carcass quality [52]. However, the effects of CSM substitution on poultry carcass characteristics remain controversial. Rao et al. [53] reported that Cobb-400 male broilers tolerated ≤15% CSM replacement during days 1–21 post-hatch, while 20% inclusion reduced slaughter performance and increased abdominal fat deposition. Conversely, Safari et al. [43] observed no impact on breast or thigh muscle weights with 20% CSM substitution in Ross 308 broilers; conversely, 30% CSM inclusion significantly decreased breast muscle mass without affecting thigh yield. Abdallh et al. [20,21] demonstrated enhanced absolute and relative thigh muscle weights in Ross 308 males with graded CSM inclusion (6%, 12%, 18%). Nevertheless, Yu et al. [54] reported controversial results, showing non-significant alterations in slaughter performance in goslings (29–63 d) fed with various proportions of SBM replacement (0–100% CSM). Our findings revealed a reduced semi-eviscerated yield, which was associated with final body weight variations. However, key parameters, such as dressing percentage, eviscerated carcass yield, breast muscle yield, thigh muscle yield, and abdominal fat percentage, remained comparable across groups. This suggests that high-protein CSM and DPCSM substitution in yellow-feathered broiler diets maintains normal body composition. The observed stability in muscle deposition may reflect improved processing techniques or compensatory metabolic adaptations. Nonetheless, further investigation is required to elucidate the underlying mechanisms.

Meat quality parameters, including color attributes (L*, a*, b*), pH, drip loss, cooking loss, shear force, and moisture content, determine the economic value of broiler production. Colorimetric indices provide direct visual assessments. Notably, L* (lightness) indicates surface reflectance, a* (redness) correlates with myoglobin content, and b* (yellowness) indicates freshness. Optimal meat quality is characterized by elevated a* and reduced L*/b* values. Postmortem pH serves as a biochemical marker of glycogenolysis intensity, with higher pH values reflecting slower anaerobic glycolysis and improved water retention, thereby enhancing meat preservation [55,56]. Shear force, a widely validated objective measure inversely correlated with tenderness [57], serves as a primary indicator of textural attributes. While sensory tenderness was not directly assessed in this study, shear force values provide a reliable proxy for mechanical resistance to mastication. Cooking/drip losses indicate the water-holding capacity and structural integrity of muscle proteins [58]. Nevertheless, studies have reported inconsistent results regarding the impacts of cottonseed meal (CSM) inclusion on meat characteristics. Abdallh et al. [59] reported enhanced color stability, notably L and a*, in CSM-fed broilers during fresh and stored phases. Islam et al. [60] observed progressive increases in crude protein, fat, and fiber content of breast muscle with graded SBM replacement (10–50% CSM) in Cobb-500 broilers, though ash content declined. In contrast, Yu et al. [54,61] reported linear reductions in linolenic acid and Σn-3 PUFA concentrations, coupled with increased Σn-6/Σn-3 ratios in goose breast muscle following CSM substitution (free gossypol: 0–183 mg/kg). While total amino acids remained stable, compositional shifts were observed in threonine, cysteine, and valine levels. These findings suggest that CSM induces modifications in lipid and amino acid metabolism. Notably, Wang et al. [52] found that CSM inclusion (19.5–163.5 g/kg diet; FG: 20–170 mg/kg) exerted no significant effect on egg quality parameters (yolk color, shell strength, albumen height) in laying hens. In the present study, increased drip loss was observed in yellow-feathered broilers fed high-protein CSM and DPCSM diets, accompanied by elevated pH (45 min/24 h) and a* 45 min values. These changes indicate reduced tenderness but improved color retention and freshness. Importantly, gossypol residues remained undetectable in breast and thigh muscles, aligning with observations in duck [9] and goose meat [61]. The preserved food safety profile, despite metabolic alterations, highlights the complex interplay between CSM utilization and meat quality outcomes.

## 5. Conclusions

High-protein cottonseed meal and de-phenolized cottonseed meal can effectively be used to replace up to 50% of soybean meal in yellow-feathered broiler diets without inducing significant adverse effects on growth performance, nutrient metabolizability, serum biochemical parameters, carcass traits, or meat quality. However, full replacement (100% substitution) resulted in significant negative impacts across these metrics.

## Figures and Tables

**Table 1:** (**a**) Ingredients and nutrient composition of the experimental diets for the starter (1–21 d) periods. (**b**) Ingredients and nutrient composition of the experimental diets for the grower (22–42 d) periods. (**c**) Ingredients and nutrient composition of the experimental diets for the finisher (43–63 d) periods.

(**a**)					
**Items ^1^**	**Starter (1–21 days)**				
	**SBM**	**CSM_50_**	**CSM_100_**	**DPCSM_50_**	**DPCSM_100_**
Ingredients (%)					
Corn	58.7	59.35	60.14	59.35	60.14
Cottonseed meal (60.32%)		7	13.6		
De-phenolized cottonseed meal (60.86%)				7	13.6
Soybean meal (43%)	25	12.2		12.2	
Corn protein meal	5	5	5	5	5
Wheat flour	5	5	5	5	5
Chili meal					
Hydrolyzed feather meal					
Corn germ meal	1.7	6.4	10.7	6.4	10.7
CaHPO_4_	1.24	1.24	1.25	1.24	1.25
Limestone	1.33	1.39	1.44	1.39	1.44
Cottonseed oil	0.6	0.6	0.6	0.6	0.6
NaCl	0.25	0.25	0.25	0.25	0.25
Lysine	0.29	0.53	0.76	0.53	0.76
Methionine	0.17	0.2	0.23	0.2	0.23
Threonine	0.04	0.13	0.22	0.13	0.22
Premix ^2^	0.68	0.71	0.81	0.71	0.81
Nutrient levels ^3^					
ME (MJ/kg)	12.95	12.95	12.95	12.95	12.95
CP (%)	18.82	18.62	18.66	18.52	18.84
Ca (%)	0.9	0.9	0.9	0.9	0.9
P (%)	0.5	0.5	0.5	0.5	0.5
Lysine (%)	1.2	1.2	1.2	1.2	1.2
Methionine (%)	0.6	0.6	0.6	0.6	0.6
Free gossypol (mg/kg)		47.30	91.90	30.63	59.51
(**b**)					
**Items ^1^**	**Grower (22–42 days)**				
	**SBM**	**CSM_50_**	**CSM_100_**	**DPCSM_50_**	**DPCSM_100_**
Ingredients (%)					
Corn	55.6	56.17	56.7	56.17	56.7
Cottonseed meal (60.32%)		5	9.3		
De-phenolized cottonseed meal (60.86%)				5	9.3
Soybean meal (43%)	17	7.8		7.8	
Corn protein meal	5	5	5	5	5
Wheat flour	10	10	10	10	10
Chili meal	2	2	2	2	2
Hydrolyzed feather meal	1	1	1	1	1
Corn germ meal	4.4	7.7	10.3	7.7	10.3
CaHPO_4_	1.01	1.02	1.02	1.02	1.02
Limestone	1.22	1.26	1.35	1.26	1.35
Cottonseed oil	1.4	1.4	1.4	1.4	1.4
NaCl	0.2	0.2	0.2	0.2	0.2
Lysine	0.36	0.53	0.67	0.53	0.67
Methionine	0.14	0.16	0.18	0.16	0.18
Threonine	0.04	0.1	0.16	0.1	0.16
Premix ^2^	0.66	0.66	0.72	0.66	0.72
Nutrient levels ^3^					
ME (MJ/kg)	13	13	13	13	13
CP (%)	17.42	17.26	17.71	17.46	17.87
Ca (%)	0.8	0.8	0.8	0.8	0.8
P (%)	0.35	0.35	0.35	0.35	0.35
Lysine (%)	1	1	1	1	1
Methionine (%)	0.5	0.5	0.5	0.5	0.5
Free gossypol (mg/kg)		33.79	62.84	21.88	40.70
(**c**)					
**Items ^1^**	**Finisher (43–63 days)**				
	**SBM**	**CSM_50_**	**CSM_100_**	**DPCSM_50_**	**DPCSM_100_**
Ingredients (%)					
Corn	58	58.48	58.83	58.48	58.83
Cottonseed meal (60.32%)		4.8	7.5		
De-phenolized cottonseed meal (60.86%)				4.8	7.5
Soybean meal (43%)	13.8	5		5	
Corn protein meal	5	5	5	5	5
Wheat flour	10	10	10	10	10
Chili meal	2	2	2	2	2
Hydrolyzed feather meal	1.5	1.5	1.5	1.5	1.5
Corn germ meal	4.1	7.3	9.1	7.3	9.1
CaHPO_4_	0.75	0.76	0.76	0.76	0.76
Limestone	1.13	1.17	1.19	1.17	1.19
Cottonseed oil	2.4	2.4	2.4	2.4	2.4
NaCl	0.2	0.2	0.2	0.2	0.2
Lysine	0.32	0.49	0.58	0.49	0.58
Methionine	0.1	0.12	0.13	0.12	0.13
Threonine	0.04	0.1	0.13	0.1	0.13
Premix ^2^	0.66	0.68	0.68	0.68	0.68
Nutrient levels ^3^					
ME (MJ/kg)	13.05	13.05	13.05	13.05	13.05
CP (%)	17.05	17.18	16.91	16.68	16.93
Ca (%)	0.7	0.7	0.7	0.7	0.7
P (%)	0.3	0.3	0.3	0.3	0.3
Lysine (%)	0.9	0.9	0.9	0.9	0.9
Methionine (%)	0.35	0.35	0.35	0.35	0.35
Free gossypol (mg/kg)		32.44	50.68	21.00	32.82

^1^ SBM: fed a corn-soybean meal basal diet; CSM: cottonseed meal; DPCSM: de-phenolized cottonseed meal; CSM_50_, CSM_100_, DPCSM_50_, and DPCSM_100_ indicate that 50 and 100% of the protein content provided by soybean meal in the control diet was replaced by CSM or DPCSM. (4.8 and 7.5% CSM or DPCSM, respectively, was added to the diet). ^2^ The premix provides the following per kg of diet: Mn 78 mg, Zn 72 mg, Cu 8 mg, Se 0.20 mg, I 0.40 mg, vitamin A 12,000 IU, vitamin D3 2500 IU, vitamin E 15 IU, vitamin K3 2.2 mg, vitamin B12 0.02 mg, nicotinic acid 35 mg, pantothenic acid 12 mg, folic acid 1.2 mg, biotin 0.15 mg, choline chloride 1200 mg. ^3^ CP values were measured, while the other values were calculated. ME: metabolizable energy; CP: crude protein; Ca: calcium; P: total phosphorus.

**Table 2:** Effect of high-percentage replacement of SBM by high-protein CSM and DPCSM on the growth performance of yellow-feathered broilers.

Items ^1^	SBM	CSM_50_	CSM_100_	DPCSM_50_	DPCSM_100_	*p*-Value
1–21 d						
21 d WG, g	551.92 ± 3.85 ^a^	527.71 ± 12.97 ^ab^	504.68 ± 32.56 ^b^	549.86 ± 17.69 ^a^	500.70 ± 19.92 ^b^	0.004
ADFI, g	36.60 ± 1.15	35.94 ± 0.77	35.37 ± 0.80	36.11 ± 0.30	34.33 ± 1.66	0.065
ADG, g	24.33 ± 0.18 ^a^	23.18 ± 0.62 ^ab^	22.08 ± 1.55 ^b^	24.23 ± 0.84 ^a^	21.89 ± 0.95 ^b^	0.004
F/G	1.51 ± 0.04	1.55 ± 0.05	1.61 ± 0.14	1.49 ± 0.05	1.57 ± 0.13	0.370
22–42 d						
42 d WG, g	1680.61 ± 7.86 ^a^	1655.21 ± 43.08 ^a^	1554.41 ± 99.21 ^b^	1661.33 ± 20.09 ^a^	1569.39 ± 37.51 ^b^	0.010
ADFI, g	100.30 ± 0.55 ^a^	100.09 ± 1.02 ^a^	94.58 ± 1.38 ^b^	98.87 ± 0.80 ^a^	95.44 ± 2.04 ^b^	<0.001
ADG, g	53.75 ± 0.42 ^a^	53.69 ± 1.74 ^a^	49.99 ± 3.25 ^b^	52.93 ± 1.00 ^a^	50.89 ± 1.03 ^ab^	0.027
F/G	1.87 ± 0.02	1.87 ± 0.05	1.90 ± 0.13	1.87 ± 0.04	1.88 ± 0.01	0.947
43–63 d						
63 d WG, g	2890.63 ± 62.40 ^a^	2787.50 ± 31.37 ^ab^	2685.42 ± 132.05 ^b^	2793.75 ± 57.89 ^ab^	2696.88 ± 52.64 ^b^	0.010
ADFI, g	144.30 ± 2.85	142.37 ± 2.52	141.89 ± 4.40	140.91 ± 2.89	140.77 ± 2.59	0.525
ADG, g	57.62 ± 3.34	53.92 ± 1.10	53.86 ± 2.11	53.93 ± 3.62	53.69 ± 2.07	0.210
F/G	2.51 ± 0.20	2.64 ± 0.05	2.64 ± 0.02	2.62 ± 0.15	2.63 ± 0.13	0.604
1–63 d						
ADFI, g	94.25 ± 0.79 ^a^	93.50 ± 1.32 ^a^	91.16 ± 1.40 ^b^	92.92 ± 1.75 ^ab^	91.27 ± 2.00 ^b^	0.040
ADG, g	45.23 ± 0.99 ^a^	43.60 ± 0.50 ^ab^	41.97 ± 2.10 ^b^	43.69 ± 0.92 ^ab^	42.16 ± 0.83 ^b^	0.010
F/G	2.09 ± 0.06	2.15 ± 0.02	2.18 ± 0.09	2.13 ± 0.04	2.17 ± 0.05	0.235

^a, b^ Means with different superscripts within the same row differ significantly (*p* < 0.05). ^1^ WG: fasted body weight; ADG: average daily gain; ADFI: average daily feed intake; F/G: feed-to-gain ratio. SBM: fed a corn-soybean meal basal diet; CSM: cottonseed meal; DPCSM: de-phenolized cottonseed meal; CSM_50_, CSM_100_, DPCSM_50_, and DPCSM_100_ indicate that 50 and 100% of the protein content provided by soybean meal in the control diet was replaced by CSM or DPCSM (4.8 and 7.5% CSM or DPCSM, respectively, was added to the diet).

**Table 3:** Effect of high-percentage replacement of SBM by high-protein CSM and DPCSM on the apparent metabolic rate of yellow-feathered broiler diets.

Items ^1^	SBM	CSM_50_	CSM_100_	DPCSM_50_	DPCSM_100_	*p*-Value
21 d						
DM, %	75.32 ± 0.88	75.10 ± 0.96	75.10 ± 2.36	74.92 ± 2.71	75.19 ± 0.30	0.980
OM, %	75.90 ± 0.87	77.19 ± 0.85	72.81 ± 2.72	76.30 ± 1.79	74.08 ± 1.54	0.051
GE, %	77.81 ± 0.63	77.49 ± 1.03	77.76 ± 2.21	77.16 ± 2.37	77.30 ± 0.31	0.958
CP, %	68.01 ± 2.16	67.86 ± 0.87	64.98 ± 4.44	67.03 ± 3.74	68.55 ± 0.77	0.408
EE, %	77.22 ± 0.33	77.16 ± 0.33	76.95 ± 2.41	75.84 ± 3.02	74.37 ± 0.51	0.970
Ca, %	59.75 ± 2.24 ^a^	48.36 ± 2.42 ^c^	54.82 ± 4.53 ^ab^	49.68 ± 3.83 ^bc^	54.68 ± 1.85 ^ab^	0.014
P, %	48.05 ± 6.74	44.51 ± 0.63	39.89 ± 5.04	37.92 ± 5.68	43.99 ± 2.06	0.202
42 d						
DM, %	67.03 ± 0.44	68.54 ± 1.51	68.01 ± 0.34	68.69 ± 1.88	65.96 ± 1.15	0.099
OM, %	68.85 ± 1.02	64.94 ± 3.67	67.73 ± 2.20	69.40 ± 1.49	69.11 ± 2.04	0.179
GE, %	70.99 ± 0.44 ^a^	71.48 ± 1.12 ^a^	71.36 ± 0.37 ^a^	71.84 ± 1.11 ^a^	69.26 ± 1.13 ^b^	0.041
CP, %	43.95 ± 1.42	45.58 ± 1.09	45.41 ± 1.66	43.86 ± 4.17	42.59 ± 2.59	0.572
EE, %	70.88 ± 1.46 ^a^	69.58 ± 2.17 ^ab^	67.20 ± 1.44 ^bc^	67.44 ± 1.70 ^bc^	65.66 ± 1.85 ^c^	0.029
Ca, %	50.38 ± 1.51	52.60 ± 0.48	49.19 ± 1.00	45.99 ± 6.34	49.48 ± 3.31	0.258
P, %	41.55 ± 2.52 ^a^	37.63 ± 2.43 ^ab^	32.42 ± 3.25 ^b^	37.13 ± 3.90 ^ab^	41.73 ± 1.95 ^a^	0.015
63 d						
DM, %	70.23 ± 0.55	68.81 ± 0.91	68.01 ± 2.24	68.81 ± 1.44	69.53 ± 0.51	0.353
OM, %	70.25 ± 0.58	69.21 ± 0.62	69.03 ± 4.09	70.28 ± 1.24	69.52 ± 1.98	0.922
GE, %	73.35 ± 0.40	72.56 ± 0.66	71.54 ± 2.01	73.06 ± 1.20	72.94 ± 0.67	0.391
CP, %	41.50 ± 0.93	38.64 ± 3.17	38.33 ± 6.54	40.11 ± 1.85	39.63 ± 3.17	0.834
EE, %	69.62 ± 0.44	69.41 ± 0.59	69.43 ± 2.21	70.70 ± 0.79	70.89 ± 0.41	0.344
Ca, %	42.83 ± 1.03	41.48 ± 2.26	39.99 ± 4.62	41.06 ± 3.61	36.65 ± 2.73	0.225
P, %	44.76 ± 4.97	40.64 ± 4.24	40.59 ± 1.86	41.18 ± 0.64	41.02 ± 2.84	0.523

^a, b, c^ Means with different superscripts within the same row differ significantly (*p* < 0.05). ^1^ DM: dry matter; OM: crude ash; GE: gross energy; CP: crude protein; EE: crude fat; Ca: calcium; P: total phosphorus. SBM: fed a corn-soybean meal basal diet; CSM: cottonseed meal; DPCSM: de-phenolized cottonseed meal; CSM_50_, CSM_100_, DPCSM_50_, and DPCSM_100_ indicate that 50 and 100% of the protein content provided by soybean meal in the control diet was replaced by CSM or DPCSM (4.8 and 7.5% CSM or DPCSM, respectively, was added to the diet).

**Table 4:** Effect of high-percentage replacement of SBM by high-protein CSM and DPCSM on blood biochemical parameters in yellow-feathered broilers.

Items ^1^	SBM	CSM_50_	CSM_100_	DPCSM_50_	DPCSM_100_	*p*-Value
21 d						
TP, g/L	31.03 ± 2.46	30.18 ± 3.78	31.05 ± 4.33	28.83 ± 1.37	29.40 ± 2.52	0.799
ALB, g/L	12.13 ± 0.88	11.48 ± 1.12	11.35 ± 1.10	11.05 ± 0.83	11.35 ± 0.31	0.553
GLB, g/L	18.90 ± 1.64	18.70 ± 2.70	19.70 ± 3.25	17.78 ± 1.16	18.05 ± 2.25	0.788
ALT, U/L	8.25 ± 9.18	5.75 ± 0.96	3.25 ± 1.50	5.00 ± 3.83	4.50 ± 1.29	0.625
AST, U/L	199.50 ± 6.19	186.00 ± 11.02	192.50 ± 13.50	201.50 ± 26.41	189.00 ± 18.06	0.636
TG, mmol/L	1.55 ± 0.66	1.24 ± 0.60	0.93 ± 0.25	1.07 ± 0.20	1.00 ± 0.21	0.314
T-CHO, mmol/L	3.88 ± 0.47	3.10 ± 0.27	3.68 ± 0.63	3.56 ± 0.41	3.30 ± 0.53	0.218
BUN, mmol/L	0.39 ± 0.09	0.33 ± 0.08	0.40 ± 0.05	0.33 ± 0.14	0.41 ± 0.13	0.687
GLU, mmol/L	13.29 ± 0.38	13.81 ± 0.54	13.62 ± 1.51	13.34 ± 1.24	14.33 ± 0.28	0.534
42 d						
TP, g/L	33.40 ± 3.27	34.85 ± 0.70	32.83 ± 2.88	34.65 ± 3.54	36.15 ± 4.00	0.589
ALB, g/L	12.48 ± 1.09	12.35 ± 0.55	12.10 ± 0.43	12.68 ± 1.40	13.15 ± 0.93	0.607
GLB, g/L	20.93 ± 2.18	22.50 ± 0.76	20.73 ± 2.81	21.98 ± 2.51	23.00 ± 3.95	0.700
ALT, U/L	4.00 ± 1.63	4.25 ± 0.50	4.75 ± 0.96	5.00 ± 0.82	5.50 ± 1.00	0.316
AST, U/L	200.75 ± 24.46	205.75 ± 8.77	207.75 ± 26.45	200.25 ± 12.95	216.75 ± 2.22	0.683
TG, mmol/L	0.79 ± 0.24 ^b^	1.04 ± 0.33 ^ab^	0.96 ± 0.31 ^b^	1.40 ± 0.10 ^a^	0.97 ± 0.22 ^b^	0.041
T-CHO, mmol/L	3.37 ± 0.81	3.31 ± 0.44	3.51 ± 0.50	3.78 ± 0.13	4.00 ± 0.25	0.265
BUN, mmol/L	0.27 ± 0.07 ^b^	0.20 ± 0.11 ^b^	0.39 ± 0.05 ^a^	0.26 ± 0.06 ^b^	0.40 ± 0.05 ^a^	0.005
GLU, mmol/L	14.99 ± 0.78	13.62 ± 0.49	14.60 ± 0.78	14.31 ± 0.82	14.12 ± 1.17	0.244
63 d						
TP, g/L	34.80 ± 1.67	36.17 ± 2.41	33.87 ± 3.86	33.43 ± 4.08	33.13 ± 2.73	0.414
ALB, g/L	12.75 ± 0.69	12.87 ± 0.74	12.20 ± 1.02	11.88 ± 0.91	12.30 ± 0.96	0.298
GLB, g/L	22.05 ± 1.69	23.30 ± 2.37	21.67 ± 2.52	21.55 ± 3.31	20.83 ± 1.83	0.508
ALT, U/L	3.83 ± 4.02	3.33 ± 0.52	2.83 ± 1.47	2.83 ± 0.98	3.00 ± 1.67	0.912
AST, U/L	274.00 ± 51.69	241.33 ± 11.29	252.17 ± 20.60	235.33 ± 31.70	239.67 ± 25.87	0.238
TG, mmol/L	0.66 ± 0.23 ^b^	0.86 ± 0.43 ^ab^	0.68 ± 0.18 ^b^	0.92 ± 0.34 ^ab^	1.18 ± 0.27 ^a^	0.038
T-CHO, mmol/L	3.35 ± 0.29	3.53 ± 0.41	3.23 ± 0.28	2.91 ± 0.61	3.30 ± 0.41	0.178
BUN, mmol/L	0.36 ± 0.08	0.36 ± 0.10	0.43 ± 0.14	0.29 ± 0.13	0.36 ± 0.08	0.337
GLU, mmol/L	10.21 ± 0.96 ^b^	13.87 ± 1.92 ^a^	13.54 ± 1.41 ^a^	14.74 ± 2.03 ^a^	13.60 ± 1.41 ^a^	0.001

^a, b^ Means with different superscripts within the same row differ significantly (*p* < 0.05). ^1^ TP: total protein; ALB: albumin; GLB: globulin; BUN: urea nitrogen; TG: triglycerides; T-CHO: total cholesterol; ALT: alanine aminotransferase; AST: aspartate aminotransferase; GLU: glucose. SBM: fed a corn-soybean meal basal diet; CSM: cottonseed meal; DPCSM: de-phenolized cottonseed meal; CSM_50_, CSM_100_, DPCSM_50_, and DPCSM_100_ indicate that 50 and 100% of the protein content provided by soybean meal in the control diet was replaced by CSM or DPCSM (4.8 and 7.5% CSM or DPCSM, respectively, was added to the diet).

**Table 5:** Effect of high-percentage replacement of SBM by high-protein CSM and DPCSM on slaughter performance of yellow-feathered broilers.

Items ^1^	SBM	CSM_50_	CSM_100_	DPCSM_50_	DPCSM_100_	*p*-Value
Dressing percentage, %	92.31 ± 0.94	91.89 ± 0.88	92.10 ± 0.88	92.01 ± 0.76	91.71 ± 0.80	0.548
Semi-eviscerated carcass yield, %	84.06 ± 1.32 ^a^	81.09 ± 4.58 ^b^	82.79 ± 1.26 ^ab^	82.58 ± 0.89 ^ab^	81.80 ± 1.06 ^b^	0.047
Eviscerated carcass yield, %	70.61 ± 2.70	70.18 ± 4.24	67.98 ± 3.07	69.06 ± 1.13	69.06 ± 1.50	0.203
Breast muscle yield, %	20.72 ± 2.75	20.62 ± 1.96	18.71 ± 1.14	19.44 ± 1.64	20.02 ± 1.92	0.101
Thigh muscle yield, %	24.45 ± 1.64	24.23 ± 1.20	25.05 ± 1.80	24.49 ± 2.09	25.37 ± 2.08	0.552
Abdominal fat percentage, %	2.81 ± 0.72	3.24 ± 0.75	3.26 ± 0.61	2.79 ± 0.76	2.92 ± 0.95	0.417

^a, b^ Means with different superscripts within the same row differ significantly (*p* < 0.05). ^1^ SBM: fed a corn-soybean meal basal diet; CSM: cottonseed meal; DPCSM: de-phenolized cottonseed meal; CSM_50_, CSM_100_, DPCSM_50_, and DPCSM_100_ indicate that 50 and 100% of the protein content provided by soybean meal in the control diet was replaced by CSM or DPCSM (4.8 and 7.5% CSM or DPCSM, respectively, was added to the diet).

**Table 6:** Effect of feeding high-protein CSM and cottonseed protein replacement in SBM diets on meat quality of yellow-feathered broilers.

Items ^1^	SBM	CSM_50_	CSM_100_	DPCSM_50_	DPCSM_100_	*p*-Value
Drip loss, %	7.47 ± 1.86 ^b^	8.52 ± 2.81 ^ab^	10.25 ± 1.42 ^a^	8.84 ± 2.12 ^ab^	7.68 ± 1.51 ^b^	0.017
Cooking loss, %	9.51 ± 2.53	10.35 ± 2.45	12.40 ± 4.20	11.32 ± 3.22	11.99 ± 3.50	0.225
Shear force, N	37.30 ± 13.68	39.20 ± 9.66	40.83 ± 13.94	37.88 ± 10.63	52.59 ± 29.98	0.224
pH_45min_	5.52 ± 0.22 ^b^	5.44 ± 0.19 ^b^	5.63 ± 0.20 ^b^	6.09 ± 0.33 ^a^	5.94 ± 0.07 ^a^	<0.001
pH_24h_	5.39 ± 0.20 ^c^	5.35 ± 0.10 ^c^	5.36 ± 0.15 ^c^	5.65 ± 0.39 ^b^	5.86 ± 0.26 ^a^	<0.001
L*_45min_	43.56 ± 2.39	44.28 ± 1.48	43.17 ± 1.93	44.28 ± 1.74	43.62 ± 1.74	0.575
a*_45min_	2.35 ± 1.19 ^ab^	1.66 ± 0.84 ^b^	1.53 ± 0.72 ^b^	4.90 ± 6.14 ^a^	4.23 ± 3.24 ^ab^	0.049
b*_45min_	9.67 ± 1.06	8.78 ± 1.35	9.68 ± 2.21	10.25 ± 1.31	10.61 ± 1.70	0.090
L*_24h_	49.40 ± 2.98	50.00 ± 3.56	49.36 ± 1.60	50.36 ± 2.77	51.07 ± 2.77	0.589
a*_24h_	2.69 ± 1.03 ^b^	2.62 ± 1.21 ^b^	4.15 ± 1.35 ^a^	2.59 ± 1.12 ^b^	3.34 ± 1.60 ^ab^	0.024
b*_24h_	11.31 ± 2.58	11.67 ± 3.53	15.35 ± 9.62	12.09 ± 1.95	13.28 ± 2.22	0.313

^a, b, c^ Means with different superscripts within the same row differ significantly (*p* < 0.05). ^1^ L*: lightness; a*: redness; b*: yellowness. SBM: fed a corn-soybean meal basal diet; CSM: cottonseed meal; DPCSM: de-phenolized cottonseed meal; CSM_50_, CSM_100_, DPCSM_50_, and DPCSM_100_ indicate that 50 and 100% of the protein content provided by soybean meal in the control diet was replaced by CSM or DPCSM (4.8 and 7.5% CSM or DPCSM, respectively, was added to the diet).

## Data Availability

The datasets of the current study and the models used are available from the corresponding author upon reasonable request.
